# Characteristics of T cell receptor repertoires of patients with acute myocardial infarction through high-throughput sequencing

**DOI:** 10.1186/s12967-019-1768-8

**Published:** 2019-01-11

**Authors:** Zhixiong Zhong, Heming Wu, Qifeng Zhang, Wei Zhong, Pingsen Zhao

**Affiliations:** 1 Center for Cardiovascular Diseases, Meizhou People’s Hospital (Huangtang Hospital), Meizhou Academy of Medical Sciences, Meizhou Hospital Affiliated to Sun Yat-sen University, Meizhou, 514031 People’s Republic of China; 2 Clinical Core Laboratory, Center for Precision Medicine, Guangdong Provincial Engineering and Technology Research Center for Clinical Molecular Diagnostics and Antibody Therapeutics, Guangdong Provincial Key Laboratory of Precision Medicine and Clinical Translational Research of Hakka Population, Meizhou People’s Hospital (Huangtang Hospital), Meizhou Academy of Medical Sciences, Meizhou Hospital Affiliated to Sun Yat-sen University, Meizhou, 514031 People’s Republic of China; 3 Guangdong Provincial Engineering and Technology Research Center for Molecular Diagnostics of Cardiovascular Diseases, Meizhou People’s Hospital (Huangtang Hospital), Meizhou Academy of Medical Sciences, Meizhou Hospital Affiliated to Sun Yat-sen University, Meizhou, 514031 People’s Republic of China; 4 Meizhou Municipal Engineering and Technology Research Center for Molecular Diagnostics of Cardiovascular Diseases, Meizhou, 514031 People’s Republic of China; 5 Meizhou Municipal Engineering and Technology Research Center for Molecular Diagnostics of Major Genetic Disorders, Meizhou, 514031 People’s Republic of China

**Keywords:** T-cell receptor repertoires, Acute myocardial infarction, Immune response, High-throughput sequencing

## Abstract

**Background:**

T cells are key regulators of immunity and one of the cells recruited in atherosclerosis and participated in various stages of the development of atherosclerosis. Characterizing T-cell receptor (TCR) repertoires is a priority of great scientific interest and potential clinical utility for the early diagnosis, risk stratification and prognostic evaluation of acute myocardial infarction (AMI).

**Methods:**

The TCR repertoires in 21 subjects including 7 patients with non-ST-segment elevation myocardial infarction (NSTEMI), 6 patients with ST-segment elevation myocardial infarction (STEMI) and 8 subjects with normal coronary artery (NCA) as control were characterized by using high-throughput sequencing. Bioinformatics analysis were performed.

**Results:**

Patients with NSTEMI displayed more diverse TCR sequences than NCA controls, but they had lower percentage of top 200 TCR sequences. However, no significant differences were observed between the patients with STEMI and NCA controls, but STEMI group had lower percentage of top 200 TCR sequences. T cells from patients with AMI and NCA controls showed a differential V and J gene usage, especially, significant difference was observed in frequencies of V gene (TRBV2, TRBV29-1, TRBV30 and TRBV12-3) and J gene (TRBJ2-1) usage. Furthermore, significantly differences in average overlap was observed in groups of AMI and NCA control. The results showed that patients with AMI had distinct TCR repertoires which revealed the association between cardiovascular condition and T-cell clonotypes.

**Conclusions:**

Our findings revealed the differences of TCR repertoires between patients with AMI and NCA controls, which might be potential biomarkers for evaluating risk stratification or diagnosis of acute coronary syndrome.

**Electronic supplementary material:**

The online version of this article (10.1186/s12967-019-1768-8) contains supplementary material, which is available to authorized users.

## Background

Coronary artery disease (CAD) is the major cause of death and disability in the world, especially in developing countries in recent years [1]. According to clinical symptoms, extent of arterial occlusion and myocardial injury, CAD is divided into different categories, i.e. stable angina pectoris (SA), unstable angina pectoris (UA), and acute myocardial infarction (AMI). UA and AMI are also named acute coronary syndrome (ACS) [2, 3].

Although the prognosis and cardiac function of patients with acute myocardial infarction (AMI) have been greatly improved by strategies of early and effective primary percutaneous coronary intervention with thrombolysis, many patients with AMI still develop to heart failure, poses a great threat to human health, accounting for 7 and 5% of the global burden of disease in males and females, respectively [4–6]. It is well known that the rupture or erosion of atherosclerotic plaques is the main cause of acute coronary syndrome (ACS). Thus, it is critical to find circulating biomarkers for early diagnosis, risk stratification and prognostic evaluation of AMI. In our previous studies, we characterized the circulating microRNAs and long non-coding RNAs of patients with AMI. We found that some microRNAs and long non-coding RNAs might be potential biomarkers for early diagnosis and risk stratification of AMI [7, 8].

Immune condition including innate and adaptive immunity responses contributed to the development of the atherosclerosis and its complications, but the mechanisms have not yet been fully elucidated [9]. T-lymphocytes play an important role in human adaptive immune responses and recognize antigen peptides via specific T-cell receptors (TCRs) on the cell surface [10]. T-cell receptor (TCR) is a kind of molecule existing on the surface of T lymphocytes, responsible for recognizing fragments of antigen as peptides bound to major histocompatibility complex (MHC) molecules [11, 12]. TCR has been found to affect a wide range of diseases, including malignancy, autoimmune disorders and infectious diseases.

T cells are the main components of cell mediated immune response and have been demonstrated to be involved in the etiology and development of atherosclerotic plaques [13]. Adaptive T-lymphocytes driven immune inflammatory response is involved in atherosclerosis and plaque instability [14]. A large number of T lymphocytes and macrophages are found in the intimal lesions in stages of disease progression, whereas B lymphocytes and plasma cells mainly detected in the outer membrane, adjacent to late stage plaques [15, 16]. T cells are key regulators of immunity and one of the cells recruited in atherosclerosis and participated in various stages of the development of atherosclerosis [17–20]. The presence of activated T-lymphocytes within atherosclerotic lesions suggested the involvement of antigen driven immunological mechanisms in the onset and progression of AMI [21, 22].

Most TCRs consist of α- and β-chains, while others include γ-and δ-chains. These TCRs are able to specifically recognize numerous diverse peptides presented on MHC (pMHC) complexes via complementarity-determining regions (CDRs), especially CDR3. Characterizing TCR repertoires of patients with different diseases or conditions is a priority of great scientific interest and potential clinical utility, because the diversity of the TCR repertoire mirrors the human immune system [23]. Analysis of CDR3 diversity within the TCRs is crucial for understanding the basic molecular mechanisms of adaptive immunity in health and disease. Researchers are currently attempting to identify biomarkers or prognostic factors in the T-cell receptor repertoire to facilitate the early detection, treatment and prognosis of patients with cancers or AMI [24–27]. However, TCR repertoires in patients with different risks of cardiovascular diseases remains largely unknown.

In this study, TCR repertoires in subjects with AMI or NCA were characterized by using high-throughput sequencing and bioinformatics analysis were performed. The results revealed the distinct TCR repertoires in AMI patients and demonstrated the presence of disease associated to T-cell clonotypes. Our findings revealed the differences of TCR repertoires between subjects with AMI and NCA, which might be potential biomarkers for evaluating risk stratification or diagnosis.

## Methods

### Subjects

A total of 13 patients with AMI (7 NSTEMI and 6 STEMI) and 8 subjects with normal coronary artery (NCA), aged from 38 to 73 years, were enrolled from Center for Cardiovascular Diseases, Meizhou People’s Hospital (Huangtang Hospital), Meizhou People’s Hospital (Huangtang Hospital), Meizhou Academy of Medical Sciences, Meizhou Hospital Affiliated to Sun Yat-sen University, China from Feb. 2016 to Apr. 2017. The diagnosis was made on the basis of symptoms, dynamic changes of serum markers, dynamic electrocardiogram and coronary angiographic results. Patients with no stenosis in coronary arteries comprised the normal coronary artery (NCA) group. In the AMI group, patients had ischemic chest pain, increased values of cardiac enzymes, and dynamic ST-segment change on ECG. The patients with ST segment elevation were diagnosed as STEMI, and those without ST segment elevation were diagnosed as NSTEMI. Exclusion criterions were: impaired left ventricular ejection fraction ≤ 45%, congestive heart failure, chronic kidney or hepatic disease and malignant disease. This study was performed in accordance with the Declaration of Helsinki and approved by the Ethics Committee of the Meizhou People’s Hospital (Huangtang Hospital), Meizhou Academy of Medical Sciences, Meizhou Hospital Affiliated to Sun Yat-sen University, China. All participants provided written informed consent before enrolment in the study.

### Samples collection and sequencing

Three milliliters of blood samples for biochemistry testing were obtained from each subject. Plasma was separated and stored at − 80 °C till further analysis. Triglyceride (TG), total cholesterol [1], high density lipoprotein cholesterol (HDL-C) and low density lipoprotein cholesterol (LDL-C) were measured routinely.

Six milliliters of peripheral venous blood samples were collected from patients with AMI at the onset of symptoms and NCA controls in ethylenediaminetatraacetic acid (EDTA)-coated tubes and processed within 1 h. Peripheral blood mononuclear cells (PBMCs) from patients and NCA controls were isolated from venous blood for RNA extraction by density gradient centrifugation over Ficoll (MD Pacific Biotechnology Co., Ltd, Tianjin, China).

Total RNA was extracted from PBMCs using TRIzol reagent (Invitrogen, Carlsbad, CA, USA) according to the manufacturer’s instructions. The quantity and purity of total RNA were evaluated by Nanodrop 2000, and the RNA Nano 6000 Assay Kit of the Agilent Bioanalyzer 2100 system (Agilent Technologies, CA, USA) was used to analyze RNA integrity. And RNA was reversely transcribed into cDNA. Multiplex PCR is a PCR reaction in which two or more pairs of primers (Additional file 1: Table S1) are added to the same PCR reaction system and multiple nucleic acid fragments are amplified simultaneously.

After amplification and separation by agarose gel electrophoresis, products were purified using a QIAquick PCR Purification Kit. The final libraries were quantified by real-time quantitative PCR. Paired-end sequencing of samples was carried out with a read length of 150 bp using the Illumina HiSeq™ Xten (Novaseq) platform.

### Bioinformatics analysis

Bioinformatics analysis follow the basic flow: (1) The data obtained from the sequencing is raw reads, and determined if the sequencing data is suitable for subsequent analysis by quality control (QC); (2) intercepts through a sequence of joints, and removes low quality bases or sequences; (3) the clean reads are aligned to the reference sequence, and for reads on the alignment, the next step is to assemble to obtain specific functional regions, such as CDR3 regions (clones); (4) the clones whose quality meets the requirements will be used as core clones. Clones with more than one base with poor quality will be referenced and corrected with the core clones as a reference; (5) clones with one base difference for hierarchical clustering, there is only one base difference (mismatch) between each branch, which in turn clusters, clones with low cloning frequency are merged into the previous branch, and the topmost head sequence is retained; (6) the above cloned sequences were aligned to the V, D, J reference sequences again. The resulting statistics file contained cloned sequences, amino acid residue sequences, clone numbers, clone frequencies and V/J gene combinations. Cloning, gene recombination, diversity analysis and other in-depth excavation can be based on this information.

### Statistical analysis

SPSS statistical software version 19.0 was used for data analysis. Data were reported as the mean ± SD. T-test, Chi-square test and ANOVA test were used to analyze the differences among the groups. Statistical significance was set at a P < 0.05.

## Results

### Baseline clinical characteristics

The baseline characteristics, medications and laboratory data of the AMI patients and NCA controls were listed in Table 1. There were significantly differences both the number of smokers and the history of clopidogrel used in the three groups. There were no significantly differences in the aspects of age, sex, triglycerides (TG), cholesterol, high-density lipoprotein cholesterol (HDL-C), low-density lipoprotein cholesterol (LDL-C), serum troponin values (cTnI), B-type natriuretic peptide (BNP), CD3+ T lymphocyte cell count, clinical drug of taking aspirin, statins, angiotensin-converting enzyme inhibitors/angiotensin antibody, Ca^2+^ antagonist and β-blocker between the AMI patients and NCA controls. The accompanied diseases in NCA group included hypertension and hyperlipidemia. Acute myocardial infarction (AMI) was the main disease in NSTEMI and STEMI groups, which accompanied diseases in these two groups included arrhythmia, hypertension and hyperlipidemia.

**Table 1:** The baseline clinical characteristics

Indexes	NCA (n = 8)	NSTEMI (n = 7)	STEMI (n = 6)	P value
Age, years	53.75 ± 5.90	58.43 ± 6.19	53.50 ± 8.55	0.339
Sex (F/M)	5/3	6/1	4/2	0.583
Risk factor				
Smoker	0	4	3	0.038
Triglycerides, mmol/L	1.63 ± 0.82	2.16 ± 1.23	2.24 ± 0.97	0.469
Cholesterol, mmol/L	4.66 ± 1.35	5.42 ± 1.59	5.91 ± 1.40	0.291
HDL, mmol/L	1.28 ± 0.30	1.03 ± 0.30	1.41 ± 0.25	0.072
LDL, mmol/L	2.39 ± 0.85	3.25 ± 0.93	3.24 ± 0.65	0.102
cTnI, ng/mL	0.010 ± 0.021	0.476 ± 0.282	14.533 ± 21.287	0.053
BNP, pg/mL	1350.22 ± 3391.04	4352.33 ± 6905.50	2021.00 ± 1861.27	0.447
CD3+ T lymphocyte cell count (/µL)	1438.63 ± 197.60	1509.29 ± 309.61	1362.83 ± 201.80	0.563
Clinical records				
Aspirin	2	5	1	0.080
Clopidogrel	0	5	1	0.007
Statins	0	2	1	0.283
ACEI/ARB	0	1	1	0.501
Ca^2+^ antagonist	0	0	0	NA
β-blocker	1	0	1	0.556

*ACEI/ARB* angiotensin-converting enzyme inhibitors/angiotensin antibody, *HDL* high-density lipoprotein, *LDL* low-density lipoprotein

### Analysis of the profile of TCRβ in PBMCs using high-throughput sequencing

To study the profile of the T-cell β receptor in human cells, primers were designed for multiplex PCR at the TRB V/D/J loci to amplify the CDR3 fragment at the RNA level. The PCR products were purified using magnetic beads. The enriched products were used for library construction and then sequenced at a single-base resolution.

Our study subjects included 8 NCA, 13 patients with AMI (7 patients with NSTEMI, and 6 patients with STEMI). The total number of reads was 309,908,060, with an average of 14,757,526 reads per sample. The total number of sequencing raw reads achieved from each disease group were ranging from 1.03 × 10^7^ to 2.21 × 10^7^ for NCA, 1.06 × 10^7^ to 1.59 × 10^7^ for NSTEMI, and 1.19 × 10^7^ to 1.65 × 10^7^ for STEMI, respectively; and the numbers of sequencing clean reads were ranging from 9.69 × 10^6^ to 2.15 × 10^7^ for NCA, 9.98 × 10^6^ to 1.47 × 10^7^ for NSTEMI, and 1.07 × 10^7^ to 1.53 × 10^7^ for STEMI, respectively (Additional file 2: Table S2).

### Characterization and frequency distributions of T-cell receptor in patients with AMI

**Fig. 1:**
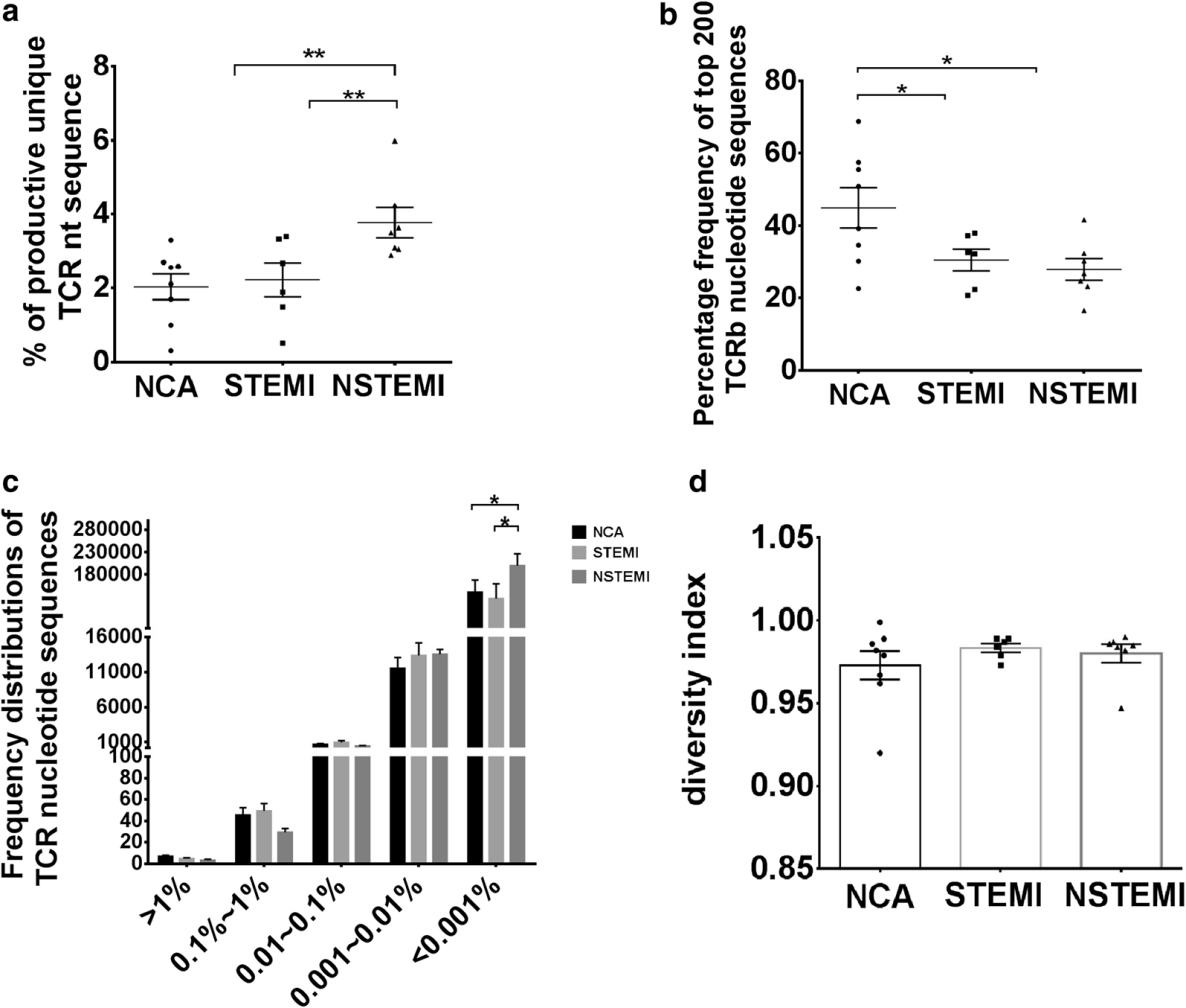
Clonal distribution of T cells in NCA controls and AMI patients. **a** Data show the percentage of productive unique TCR sequence in NCA controls and AMI patients. **b** Data show the frequency of top 200 T cell receptor repertoire nucleotide sequence in NCA controls and AMI patients. **c** Frequency distribution of TCR nucleotide sequence from NCA (n = 8) controls, NSTEMI (n = 7) and STEMI (n = 6) patients. **d** Data show the distribution of T-cell clonal frequencies through the measurement of amino acid diversity. Data are represented as mean ± SD of distribution of each patient and the bar shows the mean ± SD of the group. Each dot represents a patient’s information. The differences between groups were compared using *t*-test. **P < 0.01 and *P < 0.05. *nt* nucleotide

The number of productive unique TCR β sequences relative to the number of productive sequences provides a general assessment of diversity within a sample. The unique clonotypes of the T cells were significantly higher in the peripheral blood of NSTEMI subjects than in other two groups (NSTEMI vs. NCA, P < 0.01; NSTEMI vs. STEMI, P < 0.01) (Fig. 1a). The sum of the frequencies of the top 200 T cell clones in NCA group were significantly higher than both in STEMI group and NSTEMI group (NSTEMI vs. NCA, P < 0.05; STEMI vs. NCA, P < 0.05). The average fraction of the top 200 TCRβ sequences was 27.93% in NSTEMI, 30.52% in STEMI, and 44.92% in NCA, suggesting the TCR distribution in the NCA group was more concentrated than in the other two groups, that is, clonally expanded TCRβ nucleotide sequences in AMI patients (Fig. 1b). At the same time, our results also showed the number of T cell clones in certain frequency interval (< 0.001%) in NSTEMI groups was remarkably higher than in other two groups (NSTEMI vs. NCA, P < 0.05; NSTEMI vs. STEMI, P < 0.05), no differences were found in other frequency intervals (Fig. 1c). The clonal diversity index is one of the most important features of the T cell immune system. It reflects the immune spectrum and the function of the immune system. Our study showed no significant difference in the clonal diversity index in three groups (Fig. 1d).

### T cells from patients with AMI and NCA controls showed a differential V and J gene usage

**Fig. 2:**
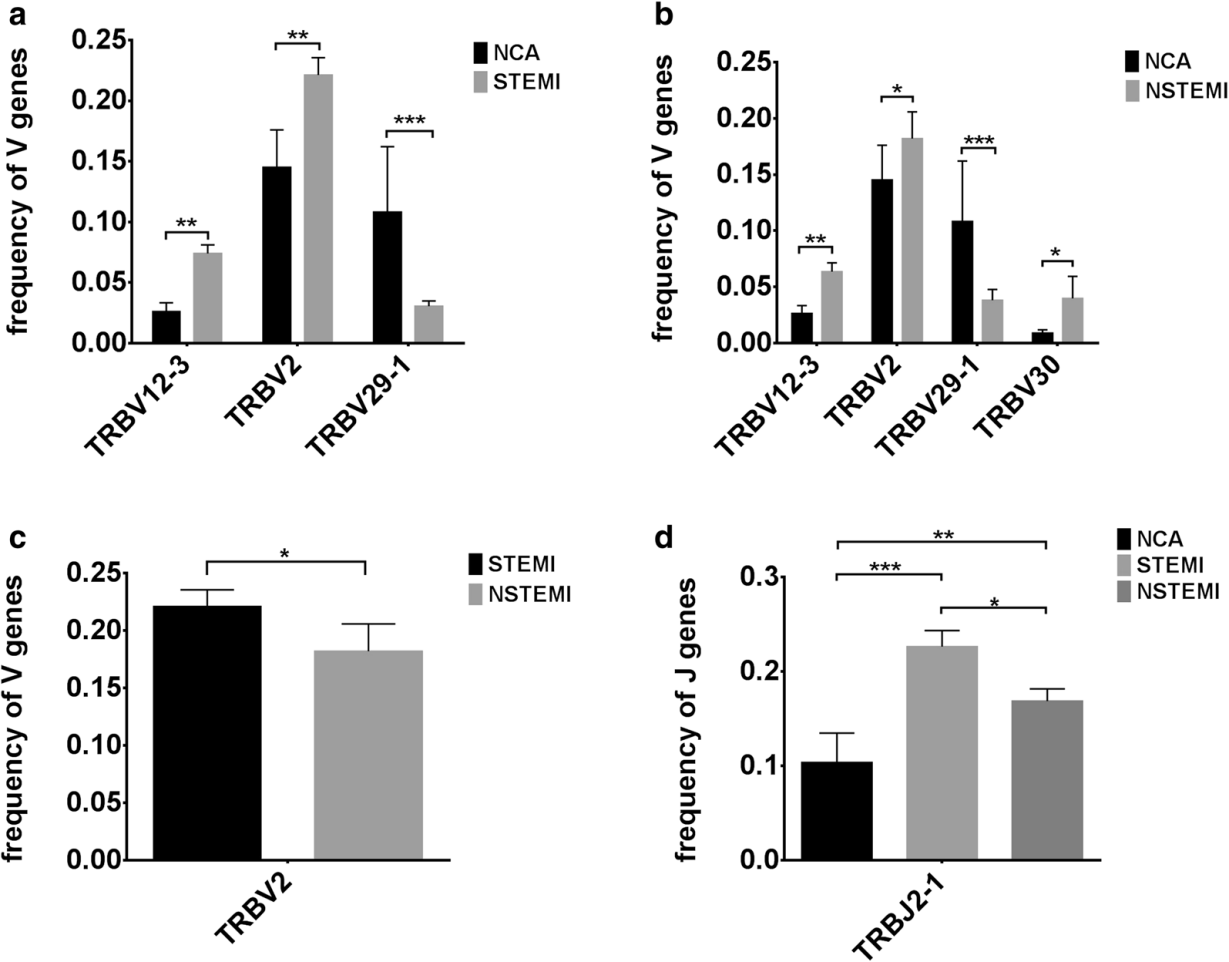
V gene and J gene usage of clonotypes in the NCA controls and AMI patients. Data show the significantly different percentage frequencies of V gene (TRBV2, TRBV29-1, TRBV30 and TRBV12-3) and J gene (TRBJ2-1) usage by clonotypes in NCA controls and AMI patients. (**a**) Frequencies of V genes usage by clonotypes in NCA controls and patients with STEMI. (**b**) Frequencies of V genes usage by clonotypes in NCA controls and patients with NSTEMI. (**c**) Frequencies of V genes usage by clonotypes in patients with STEMI or NSTEMI. (**d**) Frequencies of J genes usage by clonotypes in NCA controls and patients with STEMI or NSTEMI. Data show mean ± SD frequency of each group. Data were compared to NCA group using *t* tests. ***P < 0.001, **P < 0.01 and *P < 0.05

After identifying the sequence of VDJ genes, we obtained the expression number of each reads by counting the expression of recombinant genes, which can represent the relative number of each TRBV in statistics. The fragment abundance distributions of CDR3 V gene in all subjects show in Additional file 3: Figure S1. The average number of distinct Vβ gene segments in each sample was 45, with ranging from 42 to 48. The most frequent Vβ gene segments were TRBV2, TRBV28, TRBV29-1, TRBV20-1, TRBV11-2 and TRBV12-3. The most frequent Jβ segments were TRBJ2-7, TRBJ2-5, TRBJ2-1, TRBJ2-3, TRBJ1-1, TRBJ1-2 and TRBJ2-2. The differences of percentage frequencies of V gene (TRBV2, TRBV29-1, TRBV30 and TRBV12-3) and J gene (TRBJ2-1) usage by clonotypes in NCA controls and AMI patients were showed in Fig. 2. Compared with NCA, STEMI group showed significantly higher percentage in the clonotypes of TRBV2 (P < 0.01) and TRBV12-3 (P < 0.01), and lower percentage in TRBV29-1 (P < 0.001) (Fig. 2a), whereas NSTEMI group expressed notably higher percentage of clonotypes in TRBV12-3 (P < 0.01), TRBV2 (P < 0.05) and TRBV30 (P < 0.05), and lower percentage in TRBV29-1 (P < 0.001) compared to NCA (Fig. 2b). There was significant difference in percentage of clonotype TRBV2 (P < 0.05) between NSTEMI and STEMI (Fig. 2c). In NSTEMI and STEMI, the clonotype of TRBJ2-1 accounted for higher percentage compared to NCA (Fig. 2d).

**Fig. 3:**
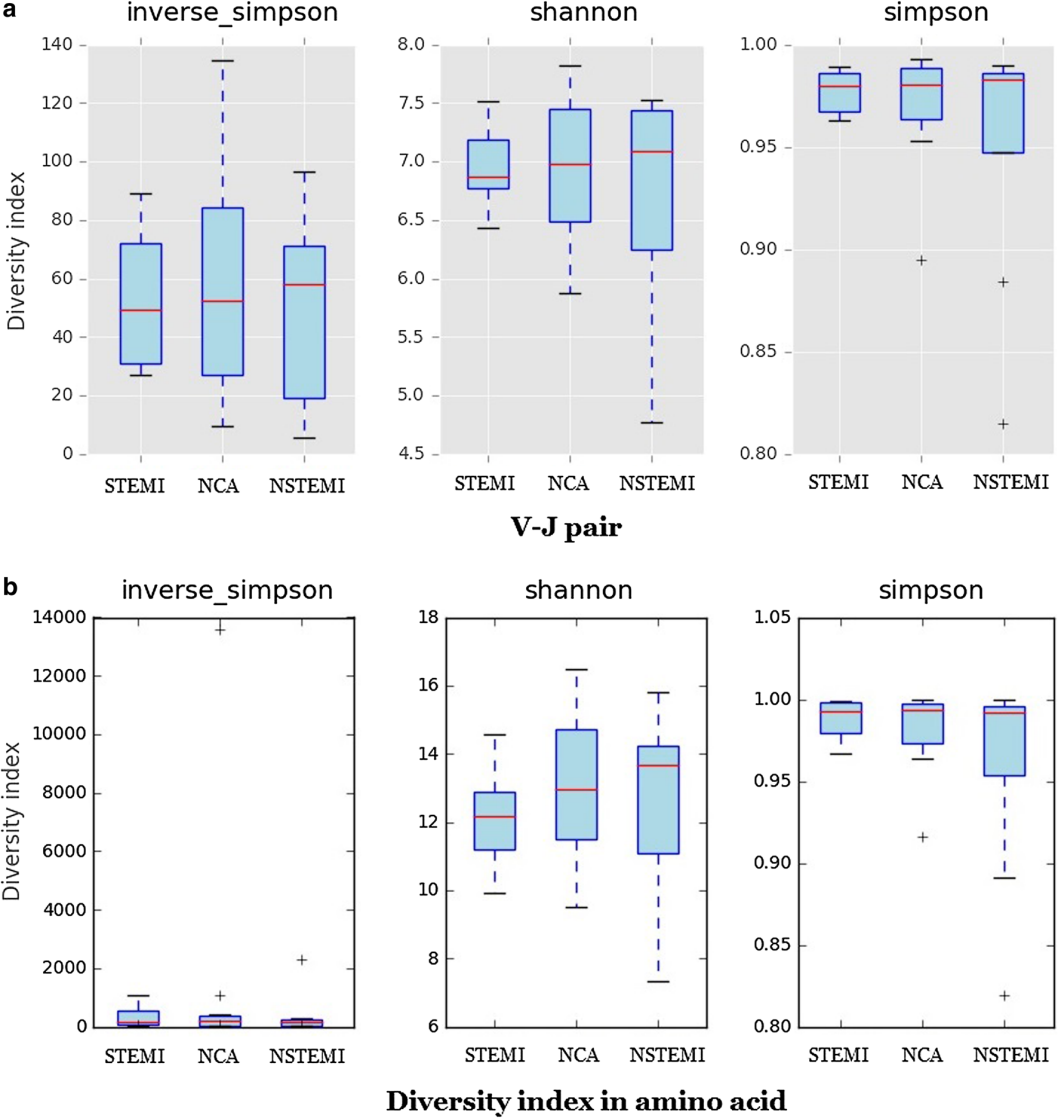
The diversity calculated by each sample is invsimpson, simpson and Shannon Weiner coefficient. The diversity index of V–J pair (**a**) and amino acid sequence (**b**) were calculated respectively

**Fig. 4:**
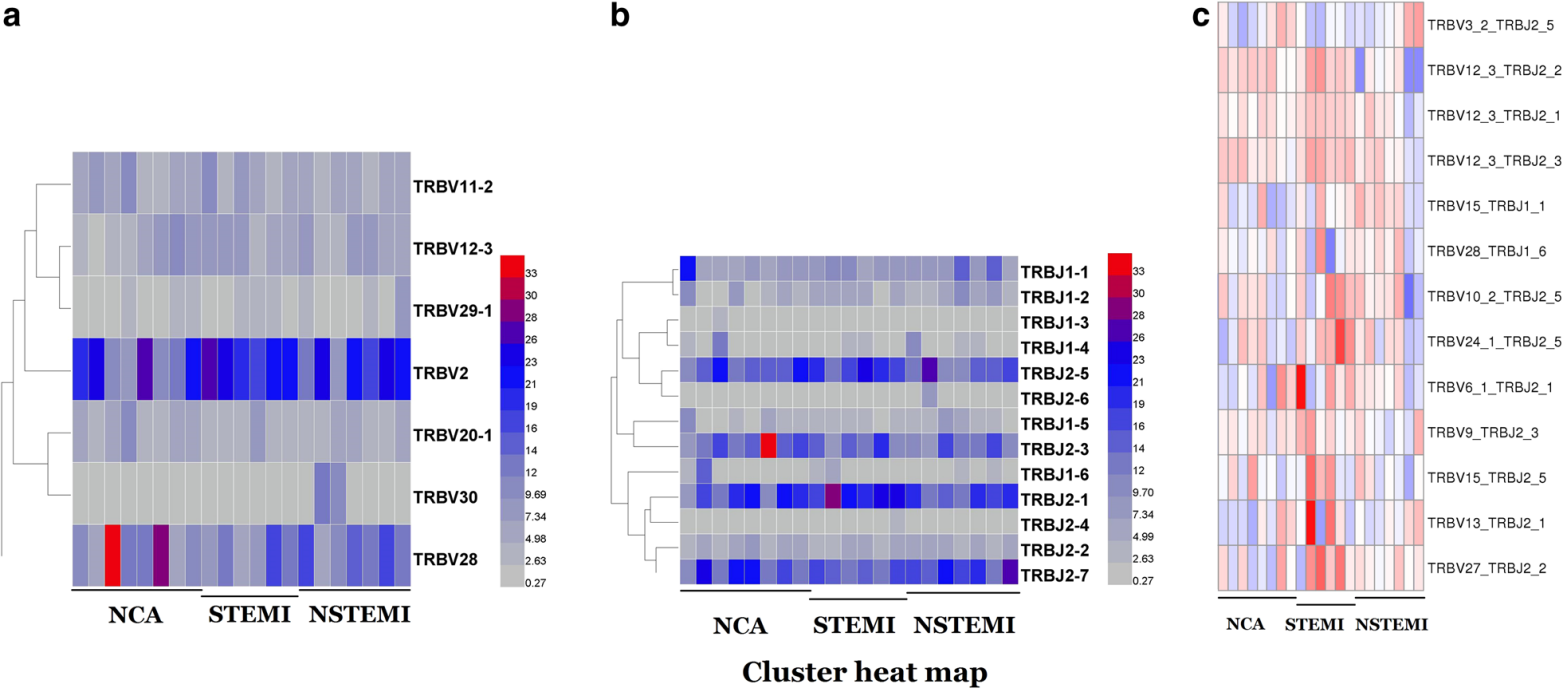
Hierarchical clustering of V and J gene usage in AMI patients and NCA controls. *NCA* normal coronary artery, *STEMI* ST-segment elevation myocardial infarction, *NSTEMI* non-ST-segment elevation myocardial infarction. **a** V gene usage; **b** J gene usage; **c** V–J pair

The diversity calculated by each sample is invsimpson, simpson and Shannon Weiner coefficient. The diversity index of V–J pair and amino acid sequences were calculated respectively (Fig. 3). Hierarchical clustering of V and J gene usage in AMI patients and NCA controls showed in Fig. 4.

### Overlap in TCRβ repertoires between individuals and groups

**Fig. 5:**
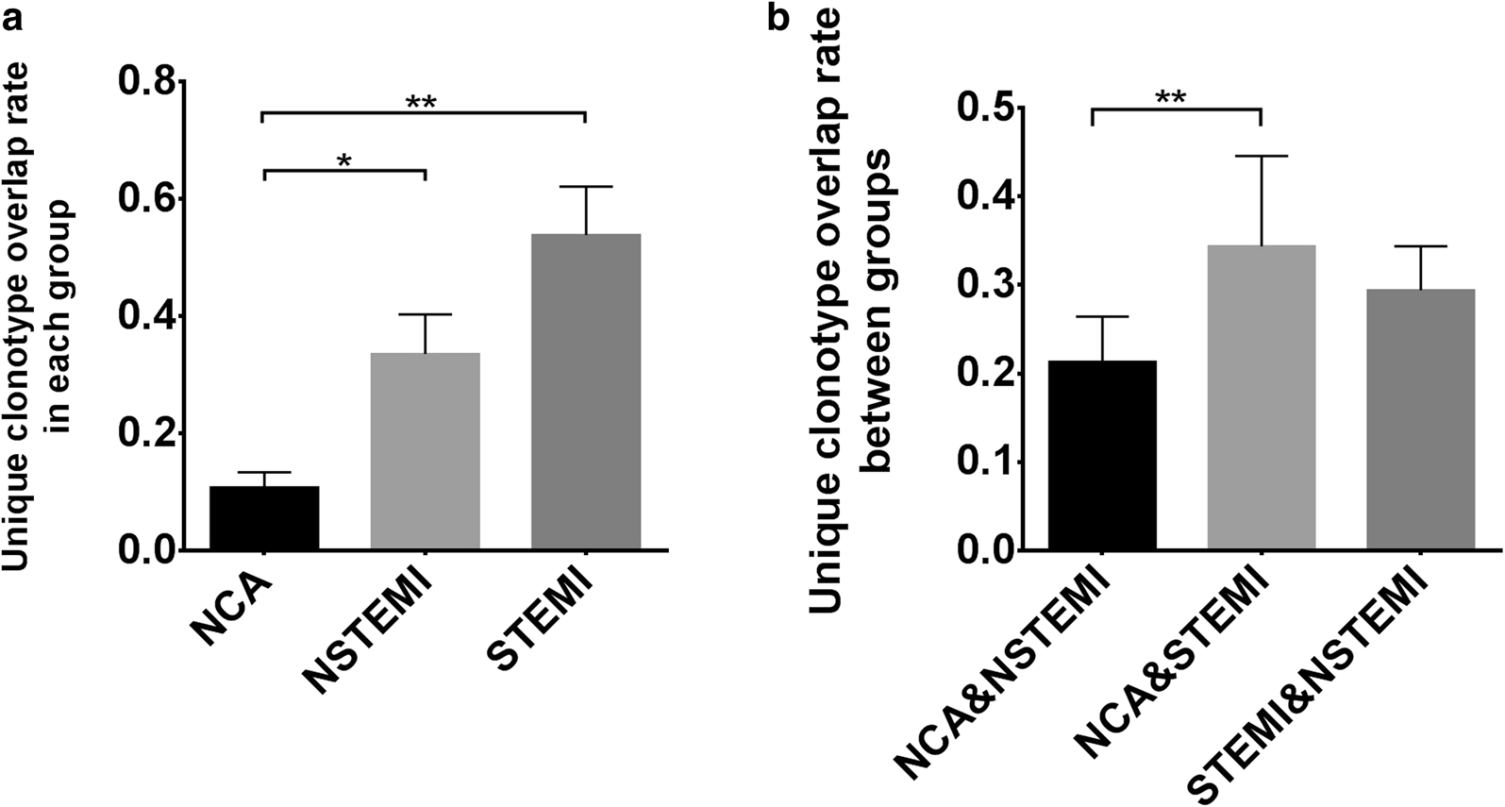
Unique clonotype overlap rate in each and between groups. **a** The data show the overlap of clonotypes in NCA, NSTEMI and STEMI group. **b** The data show the unique clonotype overlap rate between groups: NSTEMI and NCA, STEMI and NCA, STEMI and NSTEMI. Data was analysis with one-way ANOVA. **P < 0.01 and *P < 0.05

Intra-group and inter-group comparisons were made for investigated the degree of overlap in TCRβ repertoires. The results showed that the difference in average overlap in the NCA and NSTEMI groups, the NCA and STEMI groups were statistically significant (Fig. 5a). Between the NCA and STEMI samples, the overlap significantly higher than that observed between NCA and NSTEMI. No significantly difference was found between the NCA and STEMI samples compared to between the STEMI and NSTEMI samples, NCA and NSTEMI compared to between the STEMI and NSTEMI samples (Fig. 5b). The common CDR3 amino acid clonotypes among three groups showed in Table 2. As the Table 3 shown, the common CDR3 amino acid clonotypes were identified in NSTEMI and STEMI groups but not found in the NCA group.

**Table 2:** The common CDR3 amino acid clonotypes among three groups

CDR3 amino acid clonotypes	NCA group	NSTEMI group	STEMI group
CASSFTDTQYF	1.43E−05	4.83E−13	7.72E−07
CASSGTGGGETQYF	0.001495	4.54E−06	3.33E−06
CASSLAGDTQYF	1.98E−08	2.36E−08	1.33E−05
CASSLAGNTEAFF	2.86E−06	2.74E−08	5.14E−08
CASSLDRGYEQYF	4.28E−06	4.25E−07	5.88E−08
CASSLDRNYGYTF	6.04E−06	2.33E−06	1.32E−05
CASSLDSSYEQYF	1.24E−05	2.16E−13	3.80E−07
CASSLDSYEQYF	2.38E−06	3.29E−07	2.04E−06
CASSLGDTQYF	5.23E−19	3.67E−46	4.50E−13
CASSLGETQYF	1.95E−18	8.74E−40	1.42E−18
CASSLGGDTQYF	1.48E−05	5.40E−07	1.33E−05
CASSLGGSSYNEQFF	1.42E−07	2.75E−27	2.01E−13
CASSLGGSYNEQFF	8.80E−07	1.74E−26	3.25E−13
CASSLGGTDTQYF	7.93E−07	1.74E−13	9.92E−08
CASSLGGYGYTF	4.48E−06	9.16E−14	1.80E−12
CASSLGQETQYF	2.60E−06	1.88E−07	3.31E−08
CASSLGQQETQYF	7.93E−07	1.40E−13	4.00E−06
CASSLGSYEQYF	1.62E−06	3.74E−27	1.19E−14
CASSLNTGELFF	1.08E−06	3.66E−34	2.57E−08
CASSLSGSSYNEQFF	1.35E−06	2.36E−08	2.57E−08
CASSLSSGANVLTF	9.78E−07	2.68E−13	1.84E−05
CASSLTDTQYF	4.84E−13	6.38E−33	2.57E−08
CASSLTGGTEAFF	1.05E−06	4.08E−13	2.57E−08
CASSLTYEQYF	2.00E−06	3.17E−26	2.57E−08
CASSPGGETQYF	5.84E−06	4.60E−13	9.85E−07
CASSSDSYEQYF	7.99E−06	2.36E−08	2.57E−08

**Table 3:** The common CDR3 amino acid clonotypes among NSTEMI and STEMI groups but not found in the NCA group

CDR3 amino acid clonotypes	NSTEMI group	STEMI group
CASSAPNSPLHF	0.00046	3.97192E−07
CASSEGETQYF	1.01E−07	2.44906E−06
CASSESGSSTDTQYF	1.78E−06	1.21557E−12
CASSFGTDTQYF	1.13E−06	2.36608E−07
CASSFSGANVLTF	3.29E−07	1.52354E−06
CASSFSGSSYNEQFF	2.03E−08	4.90816E−06
CASSFSTDTQYF	3.31E−07	2.41368E−06
CASSGNTDTQYF	3.41E−06	1.76799E−06
CASSGTGGNQPQHF	5.25E−06	2.14629E−06
CASSHTDTQYF	2.01E−06	0.000130407
CASSLAGAGANVLTF	4.57E−07	6.75233E−06
CASSLAGANEQFF	4.06E−07	7.95009E−07
CASSLAGGPYEQYF	7.54E−07	1.28212E−06
CASSLAGGPYNEQFF	2.36E−08	7.39482E−13
CASSLAGGSSYNEQFF	1.79E−12	2.49352E−07
CASSLAGGSYNEQFF	6.34E−20	2.56952E−08
CASSLAGQETQYF	7.37E−20	2.56964E−08
CASSLAGSSYNEQFF	3.07E−07	1.77529E−06
CASSLAGYEQYF	1.25E−13	5.87587E−08
CASSLASYEQYF	5.89E−07	3.22845E−05
CASSLATDTQYF	1.21E−07	1.44169E−06
CASSLAYEQYF	9.93E−20	5.75566E−06
CASSLDGNYGYTF	1.01E−06	6.61278E−08
CASSLDQETQYF	1.51E−07	1.78166E−07
CASSLEETQYF	9.89E−20	1.42126E−06
CASSLEGYEQYF	4.79E−13	3.43803E−05
CASSLGADTQYF	1.26E−13	5.13915E−08
CASSLGAYEQYF	6.59E−07	9.62799E−06
CASSLGGAGANVLTF	6.03E−08	4.85637E−06
CASSLGGEQYF	9.62E−20	1.76276E−07
CASSLGGETQYF	4.13E−13	4.2983E−07
CASSLGGGTEAFF	1.21E−13	2.81334E−06
CASSLGGNEQFF	6.80E−26	1.98281E−14
CASSLGGNQPQHF	9.45E−14	3.18908E−06
CASSLGGNTEAFF	4.52E−07	3.01345E−14
CASSLGGQETQYF	6.11E−20	1.02786E−06
CASSLGGRETQYF	1.56E−19	6.52096E−06
CASSLGGSTDTQYF	3.29E−07	6.06088E−12
CASSLGLAGNEQFF	9.41E−07	1.80958E−05
CASSLGLAGSTDTQYF	2.77E−08	1.80009E−07
CASSLGLAGYNEQFF	0.000215	8.44536E−08
CASSLGLGETQYF	2.64E−07	3.41023E−13
CASSLGLNTEAFF	2.35E−06	1.62392E−05
CASSLGNTEAFF	7.40E−19	1.07908E−05
CASSLGQGYEQYF	1.58E−13	2.14391E−05
CASSLGQGYGYTF	3.05E−07	2.98271E−05
CASSLGQLNTEAFF	1.41E−06	0.000132057
CASSLGQNTEAFF	4.42E−13	5.9515E−07
CASSLGQNYEQYF	6.01E−07	1.39547E−05
CASSLGSGANVLTF	1.26E−05	5.139E−08
CASSLGSGSYEQYF	4.24E−06	8.10858E−07
CASSLGSNQPQHF	8.40E−14	2.44101E−06
CASSLGSQETQYF	2.17E−13	8.44536E−08
CASSLGTDYEQYF	2.75E−06	2.90042E−06
CASSLGTVNTEAFF	1.97E−06	2.56958E−08
CASSLGYEQYF	4.65E−46	5.5459E−13
CASSLGYNEQFF	1.65E−07	2.56953E−08
CASSLLGETQYF	7.05E−07	7.19765E−06
CASSLLTDTQYF	5.40E−07	1.74529E−05
CASSLNNEQFF	6.02E−07	2.56961E−08
CASSLNTEAFF	4.73E−14	3.30659E−08
CASSLQETQYF	7.02E−26	1.02498E−06
CASSLRETQYF	1.30E−19	1.11072E−11
CASSLRGNQPQHF	7.86E−07	2.61695E−07
CASSLRGNTEAFF	1.94E−13	6.57835E−06
CASSLSDTQYF	1.24E−13	3.30655E−08
CASSLSGNYGYTF	1.91E−06	5.79299E−07
CASSLSGSYNEQFF	1.88E−07	8.99292E−06
CASSLSTDTQYF	5.97E−33	1.90145E−12
CASSLTGDYGYTF	6.11E−07	2.1055E−06
CASSLVETQYF	2.63E−06	1.61606E−07
CASSLVGETQYF	3.89E−13	5.87619E−08
CASSLWSATNEKLFF	5.61E−07	2.97696E−07
CASSLYNEQFF	5.55E−20	4.45108E−06
CASSPGETQYF	5.10E−26	7.9228E−15
CASSPGGTDTQYF	8.58E−13	6.69266E−06
CASSPGLAAYEQYF	0.00055	5.46696E−07
CASSPGPYEQYF	6.33E−07	7.71334E−08
CASSPGQGPYEQYF	2.33E−06	1.90142E−06
CASSPGQGSYEQYF	5.43E−07	2.24078E−07
CASSPGQSTDTQYF	1.39E−06	7.97854E−06
CASSPGSTDTQYF	1.58E−13	0.000170974
CASSPQETQYF	6.85E−26	7.92294E−15
CASSPQGYEQYF	2.03E−07	3.04993E−06
CASSPYTDTQYF	3.01E−08	1.20776E−07
CASSQSYEQYF	1.55E−13	3.11586E−05
CASSRDGYEQYF	9.04E−08	6.59208E−05
CASSRDRNTEAFF	2.82E−07	8.30889E−08
CASSRDRQETQYF	2.67E−06	2.56949E−08
CASSRLAGGYNEQFF	1.36E−06	4.25376E−07
CASSRNTGELFF	6.40E−13	1.28212E−06
CASSRQETQYF	2.59E−06	4.67709E−06
CASSRQGNTEAFF	1.11E−06	1.35723E−06
CASSRTSGSTDTQYF	3.74E−13	1.03834E−07
CASSSANYGYTF	1.36E−06	1.32255E−07
CASSSGANVLTF	8.45E−13	1.14902E−05
CASSSGETQYF	5.92E−14	2.64512E−07
CASSSGTDTQYF	3.86E−12	1.0456E−07
CASSSNTEAFF	8.85E−07	1.41589E−05
CASSSQGYEQYF	2.03E−08	5.59013E−07
CASSSSTDTQYF	2.51E−19	6.0126E−06
CASSSSYEQYF	3.88E−21	1.13424E−19
CASSYQETQYF	1.56E−12	7.07473E−07
CATSREGGETQYF	3.55E−07	8.74253E−14

## Discussion

T cells are key regulators of immune responses in the development of many diseases [28–31]. Studies demonstrated that patients with ACS had a higher frequency of activated T-cells than stable angina, which implied a critical role of T-cells in coronary artery diseases and the recurrence of ACS [32–34]. Other studies showed a skewed T-cell differentiation was widely observed in patients with ACS [35]. Dysregulation of helper T-cells had an effect on the biological outcome of the immune response and might lead to plaque destabilization in ACS patients [36]. T-cell-mediated pathogenic immune response plays an important role in the inflammatory process during atherogenesis [37, 38].

In the present study, using the powerful IR-seq technology, we comprehensively analyzed the TCR CDR3 β repertoires of patients with AMI compared with subjects with NCA. Our finding suggested that there were differences of TCR repertoires between patients with AMI and subjects with NCA. It provides a comprehensive and high-throughput approach to understand TCR repertoires in patients in response to AMI, even in subgroup of patients with STEMI or NSTEMI. We found that the patients with NSTEMI displayed more diverse TCR sequences than NCA controls, but they had lower percentage of top 200 TCR sequences. However, no significant differences were observed between the patients with STEMI and NCA controls, but STEMI group had lower percentage of top 200 TCR sequences. It demonstrated that T cells from NCA controls and patients with AMI showed a differential V and J gene usage, especially, significant difference was observed in frequencies of V gene (TRBV2, TRBV29-1, TRBV30 and TRBV12-3) and J gene (TRBJ2-1) usage between patients with AMI and NCA controls. Furthermore, we found that there were statistically differences in average overlap inner or inter groups of AMI and NCA control.

There are few studies on the relationship between AMI and TCR. One study suggested that restrictive expression of TCR γδ repertoire and alteration expression of IL-17A gene might be related to the immune response and clinical outcome in AMI patients. The expression levels of TCR Vγ1, Vγ2, and Vγ3 subfamilies in AMI patients were significantly higher than those in healthy controls. The expression pattern was Vγ1 > Vγ2 > Vγ3 in AMI patients, while Vγ1 > Vγ3 > Vγ2 in healthy controls. The significantly restricted expression of TCR Vδ subfamilies were found in AMI patients. The expression frequencies of TCR Vδ7 and TCR Vδ6 in AMI patients were significantly lower than those in healthy controls. The high clonal expansion frequencies of the TCR Vδ8, Vδ4 and Vδ3 were determined in AMI patients. High expression of Foxp3 gene was found in AMI PBMCs, while high expression of IL-17A was found in AMI γδ+ cells [27]. There are still many unexplained discoveries in this study that need to be further studied and discussed.

AMI has been proved that it was correlated with inflammations [39–42], including had been confirmed in animal experiments [43]. What’s more, the inflammatory pathways not only regulate the plaque formation in the AMI patients, but also modulate the clinical consequences of the thrombotic complications of atherosclerosis. Some studies suggested that lymphocytes may play a key role in coronary artery instability by activating various cell types throughout the coronary circulation, proving that T lymphocytes and their products are likely to become new targets for the treatment and prevention of acute coronary syndromes [40]. Some studies suggested that the important mechanism for human umbilical cord blood mononuclear cells (HUCBC) to restrict infarct size and improve left ventricular ejection fraction is achieved by significantly limiting inflammatory cytokines and inflammatory cells in the infarcted myocardium [44], Emodin-mediated protection from acute myocardial infarction was mainly via inhibition of inflammation and apoptosis in local ischemic myocardium [45]. As the same, some studies suggested that proinflammatory gene mutations determine the risk of an individual suffering from myocardial infarction and may reduce the risk of myocardial infarction through early intervention [46].

## Conclusions

In this work, we characterized T-cell receptor repertoires of patients with AMI by high-throughput sequencing. Our findings showed that patients with AMI had distinct TCR repertoires and V and J genes which revealed the association between cardiovascular condition and T-cell clonotypes. It suggested that the profile of TCR repertoires may reflect the cardiovascular condition, which might be potential biomarkers for evaluating risk stratification or diagnosis of acute coronary syndrome.

## Additional files

**Additional file 1: Table S1.** TRB V/J sequencing primers.

**Additional file 2: Table S2.** Sequencing data output quality.

**Additional file 3: Figure S1.** The fragment abundance distributions of CDR3V gene in all subjects. After identifying the sequence of VDJ genes, we obtained the expression number of each reads by counting the expression of recombinant genes, which can represent the relative number of each TRBV in statistics. The X-axis represents the V genotype of the sample and the Y-axis represents the expression abundance of each clone.

## Abbreviations

CAD: coronary artery disease
SA: stable angina
ACS: acute coronary syndrome
UA: unstable angina
NSTEMI: non-ST-segment elevation myocardial infarction
STEMI: ST-segment elevation myocardial infarction
AMI: acute myocardial infarction
EAM: experimental autoimmune myocarditis
TCR: T-cell receptor
MHC: major histocompatibility complex
pMHC: peptide-MHC
CDRs: complementarity-determining regions
EDTA: ethylenediaminetatraacetic acid
PBMCs: peripheral blood mononuclear cells
QC: quality control
TG: triglycerides
HDL-C: high-density lipoprotein cholesterol
LDL-C: low-density lipoprotein cholesterol
