# Supplementary material for: Characteristics of T cell receptor repertoires of patients with acute myocardial infarction through high-throughput sequencing
Source: J Transl Med. 2019 Jan 11;17:21. doi: 10.1186/s12967-019-1768-8 (PMC6330436; doi:10.1186/s12967-019-1768-8)
Supplement: Supplementary file 1 — Additional file 1: Table S1. TRB V/J sequencing primers. [file 12967_2019_1768_MOESM1_ESM.docx]

Table S1 TRB V/J primers.

| TRB V Primers | | TRBV14 | GGAGGGACGTATTCTACTCTGAAGG |
| --- | --- | --- | --- |
| TRBV2 | ATTTCACTCTGAAGATCCGGTCCAC | TRBV15 | TTCTTGACATCCGCTCACCAGG |
| TRBV3-1 | AAACAGTTCCAAATCGMTTCTCAC | TRBV16 | CTGTAGCCTTGAGATCCAGGCTACGA |
| TRBV4-1/2/3 | CAAGTCGCTTCTCACCTGAATG | TRBV18 | TAGATGAGTCAGGAATGCCAAAG |
| TRBV5-1 | GCCAGTTCTCTAACTCTCGCTCT | TRBV19 | TCCTTTCCTCTCACTGTGACATCGG |
| TRBV5-4/5/6/8 | TCAGGTCGCCAGTTCCCTAAYTAT | TRBV20-1 | AACCATGCAAGCCTGACCTT |
| TRBV6-4.1 | CACGTTGGCGTCTGCTGTACCCT | TRBV24-1 | CTCCCTGTCCCTAGAGTCTGCCAT |
| TRBV6-8/5/1.2 | CAGGCTGGTGTCGGCTGCTCCCT | TRBV25-1 | GCCCTCACATACCTCTCAGTACCTC |
| TRBV6-9/7/1.1/6 | CAGGCTGGAGTCAGCTGCTCCCT | TRBV27-1 | GATCCTGGAGTCGCCCAGC |
| TRBV6-4.2 | AGTCGCTTGCTGTACCCTCTCAG | TRBV28 | ATTCTGGAGTCCGCCAGC |
| TRRBV6-2/3 | GGGGTTGGAGTCGGCTGCTCCCT | TRBV29-1 | AACTCTGACTGTGAGCAACATGAG |
| TRBV7-2/4/6/7/8 | GGGATCCGTCTCCACTCTGAMGAT | TRBV30-F5 | CAGATCAGCTCTGAGGTGCCCCA |
| TRBV7-3 | GGGATCCGTCTCTACTCTGAAGAT | TRB J Primers | |
| TRBV7-9 | GGGATCTTTCTCCACCTTGGAGAT | TRBJ1.1 | CTTACCTACAACTGTGAGTCTGGTG |
| TRBV9 | CCTGACTTGCACTCTGAACTAAACCT | TRBJ1.2 | CTTACCTACAACGGTTAACCTGGTC |
| TRBV10-1 | CCTCACTCTGGAGTCTGCTGCC | TRBJ1.3 | CTTACCTACAACAGTGAGCCAACTT |
| TRBV10-2/3 | CCTCACTCTGGAGTCMGCTACC | TRBJ1.4 | AAGACAGAGAGCTGGGTTCCACT |
| TRBV11-1/2/3 | GCAGAGAGGCTCAAAGGAGTAGACT | TRBJ1.5 | CTTACCTAGGATGGAGAGTCGAGTC |
| TRBV12-3.2/5.2 | GAAGGTGCAGCCTGCAGAACCCAG | TRBJ1.6 | CATACCTGTCACAGTGAGCCTG |
| TRBV12-3.1/4/5.1 | GAAGATCCAGCCCTCAGAACCCAG | TRBJ2.1 | CCTTCTTACCTAGCACGGTGA |
| TRBJ1.1 | CTTACCTACAACTGTGAGTCTGGTG | TRBJ2.2 | CTTACCCAGTACGGTCAGCCT |
| TRBJ1.2 | CTTACCTACAACGGTTAACCTGGTC | TRBJ2.3 | CCGCTTACCGAGCACTGTCAG |
| TRBJ1.3 | CTTACCTACAACAGTGAGCCAACTT | TRBJ2.4 | AGCACTGAGAGCCGGGTCC |
| TRBJ1.4 | AAGACAGAGAGCTGGGTTCCACT | TRBJ2.5 | CGAGCACCAGGAGCCGCGT |
| TRBJ1.5 | CTTACCTAGGATGGAGAGTCGAGTC | TRBJ2.6 | CTCGCCCAGCACGGTCAGCCT |
| TRBV13 | TCGATTCTCAGCTCAACAGTTC | TRBJ2.7 | CTTACCTGTGACCGTGAGCCTG |
