# Supplementary material for: Characteristics of T cell receptor repertoires of patients with acute myocardial infarction through high-throughput sequencing
Source: J Transl Med. 2019 Jan 11;17:21. doi: 10.1186/s12967-019-1768-8 (PMC6330436; doi:10.1186/s12967-019-1768-8)
Supplement: Supplementary file 2 — Additional file 2: Table S2. Sequencing data output quality. [file 12967_2019_1768_MOESM2_ESM.docx]

Table S2 Sequencing data output quality.

| Group | Sample name | Raw reads number | Clean reads number | Raw bases num(Gb) | Clean bases num(Gb) |
| --- | --- | --- | --- | --- | --- |
| NCA | NCA1 | 14786616 | 13592323 | 2.22 | 1.73 |
|  | NCA2 | 16324892 | 15232794 | 2.45 | 1.91 |
|  | NCA3 | 10264570 | 9693837 | 1.54 | 1.3 |
|  | NCA4 | 13377964 | 12731367 | 2.01 | 1.61 |
|  | NCA5 | 14328870 | 13619181 | 2.15 | 1.81 |
|  | NCA6 | 19706952 | 19321494 | 2.96 | 2.51 |
|  | NCA7 | 19402262 | 19024646 | 2.91 | 2.43 |
|  | NCA8 | 22143266 | 21524023 | 3.32 | 2.79 |
| NSTEMI | NSTEMI1 | 15862742 | 14715092 | 2.38 | 1.82 |
|  | NSTEMI2 | 15596324 | 14726661 | 2.34 | 1.84 |
|  | NSTEMI3 | 13527508 | 12699227 | 2.03 | 1.6 |
|  | NSTEMI4 | 12775918 | 11526327 | 1.92 | 1.44 |
|  | NSTEMI5 | 11891708 | 11004696 | 1.78 | 1.36 |
|  | NSTEMI6 | 13547858 | 12795004 | 2.03 | 1.7 |
|  | NSTEMI7 | 10615478 | 9982502 | 1.59 | 1.35 |
| STEMI | STEMI1 | 16520676 | 15276661 | 2.48 | 1.91 |
|  | STEMI2 | 15570666 | 13684567 | 2.34 | 1.74 |
|  | STEMI3 | 14610194 | 13783376 | 2.19 | 1.77 |
|  | STEMI4 | 12566388 | 11277331 | 1.88 | 1.41 |
|  | STEMI5 | 11904068 | 10664292 | 1.79 | 1.34 |
|  | STEMI6 | 14583140 | 13495151 | 2.19 | 1.71 |
