# Supplementary figures and images for: Characteristics of T cell receptor repertoires of patients with acute myocardial infarction through high-throughput sequencing
Source: J Transl Med. 2019 Jan 11;17:21. doi: 10.1186/s12967-019-1768-8 (PMC6330436; doi:10.1186/s12967-019-1768-8)

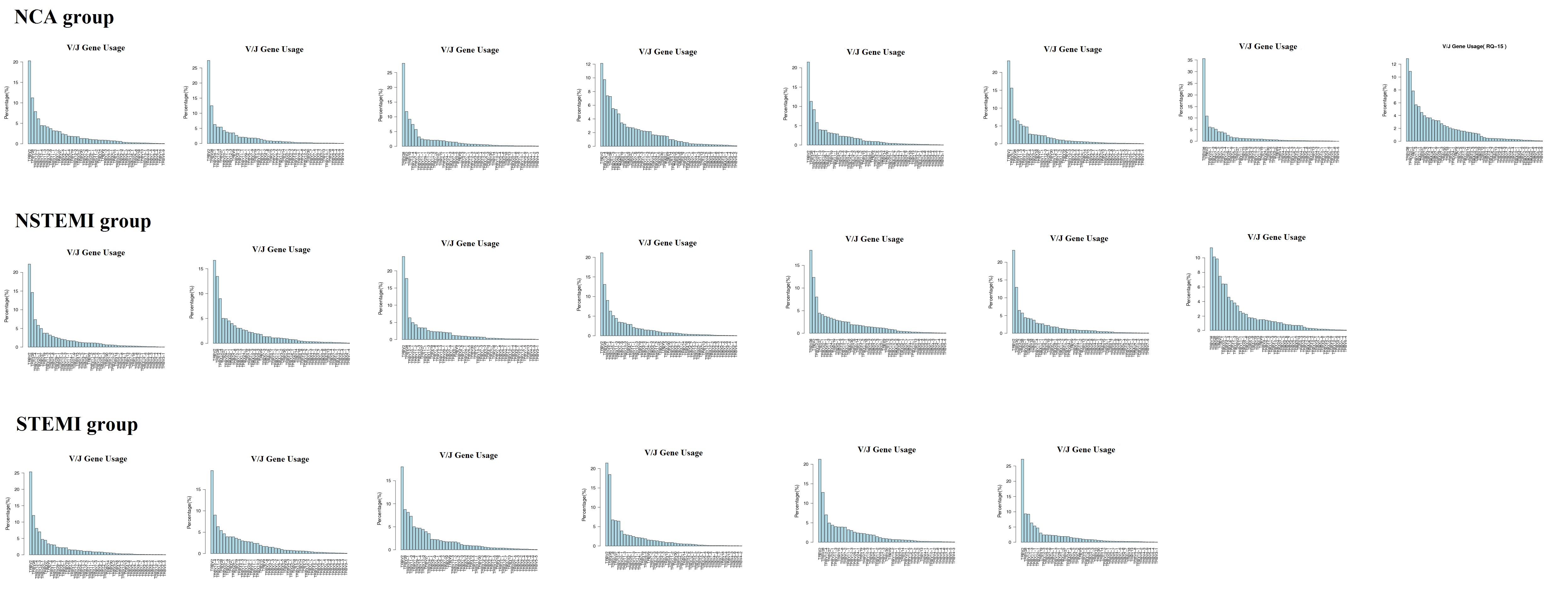

Supplement: Supplementary file 3 — Additional file 3: Figure S1. The fragment abundance distributions of CDR3V gene in all subjects. After identifying the sequence of VDJ genes, we obtained the expression number of each reads by counting the expression of recombinant genes, which can represent the relative number of each TRBV in statistics. The X-axis represents the V genotype of the sample and the Y-axis represents the expression abundance of each clone. [file 12967_2019_1768_MOESM3_ESM.jpg]
